# Mammalian Target of Rapamycin Inhibitors and Wound Healing Complications in Kidney Transplantation: Old Myths and New Realities

**DOI:** 10.1155/2022/6255339

**Published:** 2022-02-28

**Authors:** Muhammad Abdul Mabood Khalil, Saeed M. G Al-Ghamdi, Ubaidullah Shaik Dawood, Said Sayed Ahmed Khamis, Hideki Ishida, Vui Heng Chong, Jackson Tan

**Affiliations:** ^1^Department of Nephrology, RIPAS Hospital, Bandar Seri Begawan BA1710, Brunei Darussalam; ^2^Department of Medicine Faculty of Medicine, King Abdul Aziz University, Jeddah 21589, Saudi Arabia; ^3^Department of Medicine Nephrology, Division Faculty of Medicine, Menoufia University Hospital, Shibin Al Kawm, Egypt; ^4^Department of Urology &Transplant Services, Tokyo Women, s Medical University Hospital, 8-1 Kawada-cho, Shinjuku-ku, Tokyo, Japan; ^5^Department of Medicine, RIPAS Hospital, Bandar Seri Begawan BA1710, Brunei Darussalam

## Abstract

Mammalian target of rapamycin inhibitors (mTOR-I) lacks nephrotoxicity, has antineoplastic effects, and reduces viral infections in kidney transplant recipients. Earlier studies reported a significant incidence of wound healing complications and lymphocele. This resulted in the uncomfortable willingness of transplant clinicians to use these agents in the immediate posttransplant period. As evidence and experience evolved over time, much useful information became available about the optimal use of these agents. Understandably, mTOR-I effects wound healing through their antiproliferative properties. However, there are a lot of other immunological and nonimmunological factors which can also contribute to wound healing complications. These risk factors include obesity, uremia, increasing age, diabetes, smoking, alcoholism, and protein-energy malnutrition. Except for age, the rest of all these risk factors are modifiable. At the same time, mycophenolic acid derivatives, steroids, and antithymocyte globulin (ATG) have also been implicated in wound healing complications. A lot has been learnt about the optimal dose of mTOR-I and their trough levels, its combinations with other immunosuppressive medications, and patients' profile, enabling clinicians to use these agents appropriately for maximum benefits. Recent randomized control trials have further increased the confidence of clinicians to use these agents in immediate posttransplant periods.

## 1. Introduction

The combination of calcineurin inhibitor (CNI), mycophenolic acid derivatives, and steroids has reduced acute rejection by 12% in 1 year [1]. However, it has not been translated into long-term survival. Functional graft loss due to cardiovascular diseases, malignancies, and infections is still the main reason for graft loss [2–6]. Another potential reason for failure to achieve long-term survival benefits is the nephrotoxicity of the calcineurin inhibitors. mTOR-I has also been successfully used to minimize CNI in various randomized control trials. Lack of nephrotoxicity, antineoplastic, and antiviral effects make mTOR-I a good choice for transplant nephrologists to combine them with low-dose CNI [4, 6]. This maintains efficacy and reduces nephrotoxicity and viral infections in kidney transplant recipients. Unfortunately, mTOR-I's earlier use resulted in more wound healing complications and lymphocele. Wound healing complications occurred in 5–47% of the patient on sirolimus (SRL) [7, 8] and 6–40% inpatients on everolimus (EVL) [9, 10]. Incidence of lymphocele is around 4–24% in SRL [11, 12] and 7–21% in EVL [13, 14]. Wound healing complications and lymphocele formation can cause significant morbidity, longer hospital stays, more radiological investigations, and resurgical exploration or radiological intervention. This leads to an increased overall cost of transplantation. Recent randomized control trials on SRL and EVL have increased our insight into using these agents to gain maximum benefits and minimize its adverse events, including wound healing complications. Accumulating evidence has shown that other immunosuppressive medications, when used concurrently with mTOR-I, have a synergistic effect on wound healing complications. Medications implicated in wound healing complications other than mTOR-I include mycophenolic acid derivatives [15, 16], steroids [17], and ATG [18]. Similarly, various nonimmunological risk factors can also lead to wound healing complications. Nonimmunological factors include obesity, uremia, increasing age, diabetes, smoking, and protein-energy malnutrition. NEVERWOUND study is a randomized control trial that describes wound healing complications as fluid collection, including hematoma and lymphocele, prolonged lymphatic drainage (lymphorrhea), wound dehiscence, wound infection, urine leak, and incisional hernia [18]. Randomized studies which looked at these wound healing complications were thoroughly reviewed. This review focuses on the mechanism of these wound healing complications, how mTOR-I affects wound healing, risk factors for wound healing, and the way forward for optimal use of mTOR-I in light of the evidence available from randomized control trials.

## 2. Mechanism of Action of mTOR-I

The primary mechanism of action of mTOR-I is the inhibition of the mammalian target of rapamycin. It is a regulatory protein kinase involved in lymphocyte proliferation. When mTOR-I enters the cell, it binds a cytoplasmic receptor called FKBP-12. This receptor blocks serine-threonine kinase known as mTOR. This kinase (mTOR) is a downstream regulator of phosphatidyl inositol 3-kinase (PI13K) and protein kinase B (Akt). Both PI13K and AKT are activated by interleukin (IL-12, I-L15), oncogenes, vascular endothelial growth factor (VEGF), and cytomegalovirus, which activates mTOR leading to the proliferation of lymphocytes, tumor cells, cytomegalovirus, and endothelial cells. mTOR-I blocks mTOR and leads to reduced lymphocytes, endothelial cells, tumor cells, and cytomegalovirus [19, 20]. These agents cause interaction inhibition among mTOR Complex 1 (mTORC1), mTOR Complex 2 (mTORC2), and PI3K. Currently, there are two mTOR-I available. Sirolimus (SRL) is a macrolide lactone produced by *Streptomyces hygroscopicus* and has a long half-life of 62 hours. EVL is one of the derivatives of SRL and has a similar structure but has a covalently attached 2-hydroxyethyl group at position 40, leading to improved bioavailability and reducing the half-life of 26 hours [21, 22].

## 3. mTOR-I and Mechanism of Impaired Wound Healing and Lymphocele Formation

The wound healing process consists of four phases: hemostasis, inflammation, proliferation, and tissue remodeling or resolution [23]. Hemostasis consists of vascular constriction, platelet aggregation, degranulation, and fibrin formation (thrombus). The phase of inflammation includes infiltration of neutrophils, lymphocytes, and monocyte and its differentiation to macrophages. The proliferation phase includes reepithelialization, angiogenesis, collagen synthesis, and extracellular matrix formation. The final remodeling phase includes collagen remodeling, vascular maturation, and regression [24].

T cells and various cytokines play an essential role in wound healing. Various studies showed that late infiltration and reduced T cells at wound sites are associated with wound healing problems [24]. Similarly, impaired angiogenesis and reduced fibroblast activity have been implicated in wound healing [25, 26]. Vascular endothelial growth factor (VEGF) and nitrous oxide are essential mediators for angiogenesis and collagen synthesis and play a critical role in wound healing [27, 28]. mTOR-I binds FK binding protein (FKBP) and acts on the mTOR. mTOR regulates the phosphoinositide 3-kinase/Akt pathway, which is stimulated by interleukin-2 and other cytokines [29]. It also affects cell cycle progression and angiogenesis. As a result, mTOR inhibition will cause inhibition of lymphocyte, endothelial, and fibroblast proliferation. mTOR-I also causes a reduction of VEGF and NO [30]. Inhibition of endothelial and fibroblast cells by mTOR-I leads to impaired angiogenesis and fibroblastic activity [25, 31].

There are various animal studies on the pathophysiology of wound healing. It has been shown that hypoxia increases DNA synthesis and proliferative effects of platelet-derived growth factor (PDGF) and ﬁbroblast growth factor (FGF) in rat and human smooth muscle and endothelial cells. This effect is dependent on mTOR activation downstream enzyme, phosphatidyl inositol 3-kinase. Rapamycin has been shown in rats to impair wound healing by blocking this enzyme [31]. Intraepithelial lymphocytes, *γδ*T cells in the skin, help in wound healing, and depletion of these cells with rapamycin results in delayed wound healing in rats [32]. EVL has been shown to cause a reduction in hydroxyproline and collagen deposition in wounds resulting in reduced breaking strength and bursting pressure of ileal and colonic anastomosis in the rat model [33]. Similar effects were seen in the abdominal wound in rats in another study [34]. Bladder healing was assessed in rats in another study. It showed that eosinophil and neutrophil infiltration and myofibroblast proliferation were significantly lower in the bladder, fascia, and dermis of the rats who received rapamycin compared to the control group. Mean microvessel density and the percentage of cells expressing vascular endothelial growth factors in the bladder, fascia, and dermis were also significantly lower among rapamycin [35]. In a study done in pigs to assess ureteric anastomosis, the tensile strength and the hydroxyproline levels in the ureter and fascia were lower in the rapamycin-treated group [36]. Yet, in another study in pigs, although rapamycin derivatives prevented the development of bronchiolitis obliterans, it impaired the healing of bronchial anastomosis [37]. These animal studies suggest that mTOR-I impairs the ability of wound healing. Summaries of all these studies are included in Table 1.

Lymphocele *l* is a pseudocyst with lymph inside with an outside hard, fibrous capsule. It is usually adjacent to the graft [38, 39]. mTOR-I has been shown to have antilymphoangiogenic effects during surgical wound healing both in vitro and in vivo. It was demonstrated that VEGF-C plays an essential role in lymphangiogenesis. EVL and SRL inhibit this intracellular mediator of lymphangiogenesis [40].

## 4. Is It mTOR-I Only?

**Figure 1:**
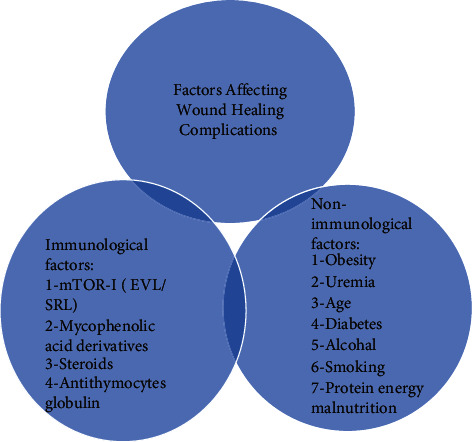
Factors associated with wound healing complications.

Besides mTOR-I, various studies conducted in animal and clinical settings have implicated other immunological medications and nonimmunological factors in wound healing complications. Therefore, it is important to investigate these factors and do a fair analysis of all these factors to reach the root cause analysis of wound healing events.  Figure 1 shows all the risk factors of wound healing complications.

These immunological and nonimmunological factors which result in impaired wound healing are as follows.

### 4.1. Mycophenolic Acid Derivatives

Mycophenolic acid derivatives include mycophenolate mofetil (MMF) and its metabolites, mycophenolic acid (MPA). It is a highly selective, noncompetitive, and reversible inhibitor of the inosine monophosphate dehydrogenase. It is the rate-limiting enzyme for de novo biosynthesis of guanosine nucleotides [41]. Guanosine nucleotides are important for DNA replication and RNA and protein synthesis. Various experimental studies have shown that MMF/MPA affects various body cells and collagen synthesis. These agents inhibit the proliferation of both *T* and *B* lymphocytes. MMF has been shown to cause downregulation of cytoskeleton proteins vinculin, actin, and tubulin in fibroblasts exposed to pharmacological doses of MPA. Skin biopsies of patients treated with MPA expressed less vinculin, actin, and tubulin than control biopsies, which could be a potential explanation for impaired wound healing [42]. Willem et al. showed in the rodent model that MMF may negatively affect the abdominal wall wound healing but had no effect on colonic anastomosis [43]. The bladder wound of rats treated with tacrolimus (TAC) and MMF has more immature collagen (type III) as compared to the control group, which has mature collagen (type I) [44]. MMF may cause inhibition of fibroblast by depletion of guanosine. Human tenon fibroblasts were cultured with various concentrations of MMF with and without guanosine, and it was shown that growth of tenon fibroblast was inhibited in a concentration-dependent way. These effects were reversed with guanosine [45]. MMF has been shown to inhibit the growth of nonimmune cells, including tubular cells [46], mesangial cells [47, 48], and myointerestitial fibroblasts [49] in the kidneys and has a potential role in proliferative glomerulonephritides and slowing down interstitial fibrosis and tubular atrophy in kidney transplant patients. In clinical studies, MMF has been implicated in causing wound healing complications in 16.6% of kidney transplant recipients [50]. In a retrospective analysis, more lymphoceles (OR = 2.6; *p* = 0.03), fluid drainage (17 vs. 5 interventions), and sclerotherapies (8 vs. 0) were observed in MMF group as compared to azathioprine (AZA) [51]. MMF has been implicated along with SRL in wound healing complications in several randomized control trials. However, it is difficult to assess the individual agent's impact on wound healing because of its use in combination with mTOR-I. In a prospective randomized control trial, hernial eventration/wound evisceration was 7/71 in the SRL-MMF group compared to 0/71 in the ciclosporin (CsA)-MMF group [52]. In the ORION study, SRL-MMF had significantly higher wound healing complications than the SRL-TAC elimination group (23% vs.16.4%, *p* < 0.05). Similarly, the incidence of lymphocele was also significantly higher in the SRL-MMF group [16]. In the SYMPHONY trial, 17% of patients had delayed wound healing in low-dose SRL and MMF groups, significantly higher than other groups (*p* value = 0.006). The incidence of lymphocele was 15.8% which was also significantly higher in the SRL-MMF group when compared to other groups (*p* value <0.001) [1]. In the TRANSFORM trial, wound healing complications were 19.8% in EVL compared to 16.2% in the MPA group, with relative risk between the two groups being 1.22 (1.01 to 1.47) [53]. In a meta-analysis of randomized control trials, the incidence of wound healing complications (OR 3.00, CI 1.61–5.59) and lymphocele (OR 2.13, CI 1.57–2.90) were significantly higher in mTOR-I and MMF as compared to mTOR-I and calcineurin inhibitor [54].  Table 2 shows the summary of experimental studies and studies conducted in a clinical setting on mycophenolic derivatives on wound healing complications.

### 4.2. Steroids

Corticosteroids cause wound healing complications by a variety of mechanisms. Corticosteroids reduce inflammation, fibroblast proliferation, collagen synthesis, angiogenesis, and reepithelialization [55]. In vitro studies conducted in an animal model have shown that steroids cause impaired wound healing through various mechanisms. Methyl prednisolone treatment has been shown to decrease transforming growth factor beta (TGF-beta) and insulin-like growth factor I (IGF-I) in the wound fluid and hydroxyproline content in the tissue (*p* < 0.05) in rats' model [56]. In another study, administration of hydrocortisone in mice reduced the skin wound healing resistance during the first postoperative week [57]. Steroids have also been implicated as risk factors in a retrospective analysis of abdominal wounds complicated by dehiscence in the general population [58].

In several randomized control trials, steroids avoidance led to fewer wound complications. Sandrini et al. showed that overall wound complications were significantly lower in the off-steroids group than those on steroids (18.8% vs. 45.6%, respectively, *p* < 0.0004). Similarly, incidence of lymphocele (5.0% vs. 32.3%, *p* < 0.0001) and dehiscence (0% vs. 10.3%, *p* < 0.009) were significantly lower in steroids avoidance group [17]. The addition of steroids to SRL increases 4.2-fold the risk for wound complications [17]. In another to randomized control trial, the incidence of lymphocele was higher in steroid-free regimens than low-dose steroids (1.5% vs. 5.9%), but it was not statistically significant [59]. Roger et al. compared 109 patients treated with a corticosteroid avoidance regimen with a historical control group (*n* = 72) that received CsA, MMF, and steroids. The corticosteroids avoidance group has lower incidence of wound healing complications (13.7% vs. 28%, *p* = 0.03) and lymphoceles (5.5% vs. 16%, *p* = 0.02) than the control group [60].

Steroids use in humans has shown that high-dose corticosteroid administration for <10 days has no clinically significant effect on wound healing. In patients taking chronic corticosteroids for at least 30 days before surgery, their rates of wound complications may be increased 2 to 5 times compared with those not taking corticosteroids [61].

### 4.3. Antithymocyte Globulin (ATG)

Various studies have also implicated ATG in wound healing problems. Benavides et al. [62] studied wound healing complications in patients receiving rabbit antithymocyte globulin (rATG) induction for a maximum of two weeks postoperatively. Patients receiving ATG: 39.1% patients have significant wound healing complications compared to 26.0% basiliximab induction (*p* = 0.025). Pourmand et al. found a significant relationship between ATG therapy and wound complications (*p* = 0.034) [63]. These findings were confirmed in the NEVERWOUND study, which reported an increased risk of wound healing >60% while using ATG induction [18].

### 4.4. Obesity

Obesity is another important risk factor accounting for wound complications. Around 34.5% of kidney transplant recipients have a body mass index (BMI) greater than 30 [64]. Wound infection and dehiscence are more when BMI is  > 30 [65]. The risk of wound healing complications goes up with the severity of obesity. Andrade et al. assessed the effect of weight on wound complications in underweight (BMI < 20 kg/m^2^), normal weight (20 ≤ BMI < 25), overweight (25 ≤ BMI < 30), class I obese (30 ≤ BMI < 35), class II obese (35 ≤ BMI < 40), and class III obese (BMI ≥ 40). There was a significantly increased risk of wound complications by 1.9-fold for every 5 points increase in BMI (*p* < 0.001), and wound complications were observed 17.5, 29.0, 45.0, and 60% with BMIs of 30, 35, 40, and 45, respectively, in each group [64]. In an analysis of data of 869 kidney transplant recipients, Lynch et al. [64] reported a graded increase in the frequency of wound infection from 8.5% among those with BMI 20–25 to 40% among those with BMI > 40 [66]. In another study conducted on SRL to assess risk factors for wound healing, obesity was an important contributor. The authors compared SRL-MMF patients with complications within three months of transplantation with SRL-MMF patients without complications and matched renal transplant recipients receiving TAC-MMF. Obesity (BMI ≥ 30 kg/m^2^) was significantly associated with wound problems. The mean BMI of SRL cases with complications was 29.9 kg/m^2^ compared to 25.4 kg/m^2^ for SRL patients without complications (*p* = 0.047). Seventy-one percent of obese SRL patients experienced complications compared with 24.3% (*p* = 0.025) of nonobese SRL patients [67]. Another retrospective analysis assessed risk factors for wound healing complications in patients receiving de novo SRL, low-dose CsA, and corticosteroid. Multivariate analysis showed that body mass index (BMI) > 26 (odds ratio 2.498, *p* = 0.027) was a significant risk factor for wound healing complications in patients taking SRL. The risk was even higher with BMI >30 (odds ratio 3.738, *p* = 0.007) [68]. In a prospective randomized trial using high-dose SRL (15–20 ng/mL), wound healing complications increased across all BMI, except patients with a BMI less than or equal to 24 kg/m^2^. In the second phase of the same trial, after excluding BMI >32 kg/m^2^ and using a low level of SRL (10 to 15 ng/mL), the complication rate in patients with BMI 28.1 to 32.0 was 33% in the SRL group as compared with 78% in phase I of the same trial [8]. Recently, TRANSFORM study excluded patients with BMI greater than 35. The mean BMI of EVL and MPA arm was 25.6 between the two groups. EVL targeting a trough concentration of 3–8 ng/ml avoided the increased rates of lymphocele seen previously, though wound healing events/complications were still slightly higher as compared to MPA (19.8% vs.16.2%) [53]. Later in-depth analysis of TRANSFORM data by Tedesco et al. found no significant association when wound healing complications were compared with mean EVL concentration during the periods from day 4 to week 4, day 4 to month 2, and day 4 to month 12 [69].

### 4.5. Uremia and Renal Dysfunction

Unlike other surgeries done on patients with normal renal functions, kidney transplant patients have preceding uremia, which has a negative impact on wound healing. There are over 100 uremic toxins in patients with end-stage renal disease [70]. It has been shown that uremia impairs fibroblast proliferation and hydroxyproline level [71–74]. Other factors that make a chronic kidney disease patient prone to impaired wound healing include uremic itch, calcemic uremic arteriopathy, malnutrition, edema, and propensity for infections [75]. Cadaveric transplantation being an unplanned event, it is always difficult to ensure adequate dialysis in the preceding past. Live transplantation being a preplanned event always provides the opportunity to provide adequate dialysis in the preceding month.

### 4.6. Age

Increasingly a greater number of elderly populations is being transplanted nowadays. Age-related skin changes affect all stages of wound healing [76]. Platelets' adherence to injured endothelium and release of various cytokines (PDGF, TGF) is enhanced in the elderly population [77]. As a result, inflammatory cells are recruited to the wound healing site. There is early infiltration of neutrophils but delayed infiltration of monocytes-macrophages compared to the young population. Macrophages played an important role in wound healing, and their late infiltration may be one reason for impaired wound healing in this population [78]. In rat models, angiogenesis is reduced in aged rats [79] and has reduced macrophage content [80]. Wound remodeling may be impaired due to reduced collagen turnover and increased fibroblast senescence [76]. There is a paucity of aging data and its effect on impaired wound healing in the kidney transplant population.

### 4.7. Diabetes

There is paucity of data on the impact of diabetes on wound healing complications in kidney transplantation. In diabetics, there is a delayed response to injury due to impaired functioning of the leukocytes and fibroblast and reduced insulin in the face of hyperglycemia [81]. Experimental studies in the acute diabetic pig model have shown that reduced insulin-like growth factors rather than hyperglycemia resulted in impaired wound healing [82]. Another in vitro study on mice showed that diabetic fibroblasts show selective impairments in cellular responses needed for tissue repair, impaired VEGF production, and impaired response to hypoxia [83]. Osmotic diuresis and catabolism associated with uncontrolled diabetes may also impair wound healing [84, 85]. Keeping these facts in mind, it is crucial to have meticulous diabetes control pre, peri, and postoperative time for better wound healing.

### 4.8. Alcohol

Both acute alcohol intoxication and chronic alcoholism impaired wound healing. Critical alcohol consumption reduces proinflammatory cytokines in the face of inflammatory challenges. It also reduces the infiltration of neutrophils and their phagocytic function at the site of inflammation. This impairs the initial inflammatory response and increases the risk of infection [86, 87]. Alcohol also affects the proliferative phase of wound healing. It has been shown in experimental studies that epithelial healing, new blood vessel formation, collagen production, and wound closure are all reduced even with a single dose of alcohol [88, 89]. Single ethanol exposure in both in vitro and in vivo settings before the injury can cause a significant decrease in wound breaking strength due to impaired fibroblast function and collagen production [90].

### 4.9. Smoking

Smoking has been shown to affect the migration of white blood cells to the site of inflammation. There is a reduced number of monocytes and macrophages at the wound sites, while the ability of neutrophils to kill bacteria is also impaired. Smoking affects lymphocytes and natural killer cells' functional ability at the site of inflammation [91, 92]. Smoking impairs epithelization and reduces the ability of fibroblasts to migrate and proliferate, resulting in an impaired proliferative phase of wound healing [91]. Nicotine causes peripheral vasoconstriction and increases the blood's viscosity through reduced fibrinolytic activity and increased platelet aggregations. Carbon monoxide in smoker binds hemoglobin more efficiently and reduces oxygen saturation. These factors result in reduced oxygen and blood supply leading to impaired wound healing [91, 93]. It has been shown that quitting smoking improves wound healing and reduces infection [94]. It is important to stop smoking six weeks before surgery, including transplantation [95].

### 4.10. Protein-Energy Malnutrition

Malnutrition of dialysis patients is multifactorial. Inadequate protein and calorie intake, loss of appetite, inflammation, loss of residual renal function, inadequate dialysis, insulin resistance, and superimposed comorbid conditions are the various causes for malnutrition [96–98]. The prevalence of malnutrition in dialysis patients has been reported between 18% and 56% [99, 100]. The recommended dietary protein intake for clinically stable maintenance hemodialysis patients is 1.2 g/kg body weight/day. At least 50% of the dietary protein should be of high biological value. Dietary protein intake for patients on peritoneal dialysis who are clinically stable is 1.2 to 1.3 g/kg body weight/day [101].

Protein is essential for wound healing, capillary formation, fibroblast proliferation, proteoglycan, and collagen synthesis. It is also necessary for optimal phagocytic activities of leukocytes. As a result, protein-energy malnutrition (PEM) results in impaired wound healing and reduced phagocytic infection [102]. Collagen is the major protein component of connective tissue and is composed primarily of glycine, proline, and hydroxyproline. Collagen synthesis requires hydroxylation of lysine and proline and cofactors such as ferrous iron and vitamin C. Impaired wound healing results from deficiencies in any of these cofactors [103]. KDOQI guidelines also suggested that nPCR should be between 1.0 and 1.2 g/kg/d, and serum albumin should be equal to or greater than 4.0 g/dL [104]. It is important to achieve these parameters before any surgical intervention to avoid wound healing complications.

## 5. Review of Randomized Control Trials on mTOR-I

In NEVERWOUND study, a randomized control trial, wound healing complications included fluid collection, including hematoma and lymphocele, prolonged lymphatic drainage (lymphorrhea), wound dehiscence, wound infection, urine leak, and incisional hernia [18]. Ueno et al. [105] described wound healing complications such as wound dehiscence, wound infection, incisional hernia, lymphorrhea, fluid collections, peri graft hematoma, and urine leak. All fluid collections were diagnosed by either ultrasound or computed tomography (CT). We reviewed all randomized control trials which looked at these wound healing complications.

With the advent of SRL in 1972 [106], SRL was being evaluated since earlier 1996 in randomized control trials [107]. Unfortunately, not all trials looked at wound healing complications or lymphocele formations as a primary or secondary outcome. Some of the initial randomized control trials reported more wound infections, wound healing complications, and lymphocele formation [108–111]. Earlier case series and retrospective data also point to wound healing complications and lymphocele formation [52, 68, 112]. Various randomized control trials from 1999 to 2017 over the last 2 decades looked at either wound healing complications or lymphocele formations [1, 7, 8, 11, 15, 16, 108–111, 113–127]. Most of the earlier trials reported a positive association of SRL with either wound healing complications or lymphocele formations. However, most of these trials used a loading dose ranging from 6 mg to 30 mg and maintained very high trough level of 10–30 ng/mL [1, 5, 7, 12, 108–111, 113, 115, 116, 118–125]. In RCT by Kandasamy et al. [116], wound healing complications were significantly reduced when loading dose was avoided in the second phase of the trial. Various randomized control trials which compared low-dose SRL with high-dose SRL reported a smaller number of wound healing complications in low-dose SRL [6, 8, 9, 12–14, 110, 113, 116, 117] numerically. Vitko et al. compared low-dose SRL (1.5 mg first dose followed by 0.5 mg once a day vs. 6 mg first dose followed by 2 mg per day) and found a significantly lower incidence of lymphocele (*p* = 0.022) [11]. In a recent RCT, SRL was used with extended-release tacrolimus (ER-TAC). SRL-ER TAC was compared with MMF-TAC. SRL level was kept at 3–5 ng/mL, and no difference was found between the two groups in terms of wound healing and lymphocele formation [127].  Table 3 shows summaries of randomized control trials which looked into wound healing complications and lymphocele formations [1, 7, 8, 11, 12, 16, 108–127].

Since earlier 2000 multiple randomized control trials were conducted on EVL [9, 10, 13, 14, 18, 69, 105, 128–139] as shown in table [4]. Various studies compared low-dose EVL (1.5 mg/day) with high-dose EVL (3 mg per day). Wound healing complications were numerically higher in the high-dose EVL group [2, 3, 8] but were not statistically significant [129, 130, 135]. In 2013, Cooper et al. [134] showed that the higher blood level of EVR (>8 ng/mL) was also associated with increased risk (HR, 1.69; 95% CI, 1.20–2.38; *p* = 0.002) of wound healing complications. Therefore, the initial dose of 1.5 mg seems safer and more reasonable than 3 mg. Most studies used level between 3–8 ng/mL [10, 14, 69, 131–133, 135, 138, 139] without any significant impact on wound healing.

It is important to consider the patient's weight, induction therapy, and combinations of other immunosuppressive medications with mTOR-I. Obesity is an important risk factor that can potentially augment mTOR-I wound healing complications. BMI of >26 has significantly been associated with wound healing complications in patients taking SRL (odds ratio 2.498, *p* = 0.027). The risk is even larger if BMI >30 (odds ratio 3.738, *p* = 0.007) [68]. The risk increases by 1.9-fold for every 5 points of BMI across a range of BMI from 20 to greater than 40 BMI in kidney transplant recipients [64]. In a systemic approach to minimize wound healing complications, multivariate analysis of recipients treated with de novo SRL showed that a BMI more than 30 to 32 kg/m^2^ was the most significant variable related to delayed wound healing (OR 3.01, 0.02) and the need to repair a transplant wound surgically (OR 8.05, *p* = 0.0001) [140]. Kandasamy et al., in the second phase of their trial, showed that exclusion of BMI >32 significantly reduced wound healing complications in SRL groups [116]. In the NEVERWOUND study, BMI of <25 kg/m^2^ was identified as a predictor of WHC-free status at 12 months [18]. Besides, consideration of obesity induction with ATG and subsequent use of mTOR-I also increase wound healing complications [18, 62, 63]. Another important fact is that using SRL and MMF may have a synergetic effect on wound healing complications [1, 16, 53, 54]. However, few studies on EVL combined with MPS or MMF did not show this synergism. de Fijter et al. [9] compared EVL-MPS with EVL-CNI and found no difference in wound healing complications. Similarly, in the CALLISTO study, no difference was observed in the incidence or severity of wound healing complications in kidney transplant recipients receiving either MMF or EVR as de novo immunosuppressive drug [10]. Nashan et al. [137] also compared EVL-MPS with EVL-CNI and found no difference in wound healing complications in BMI category ≤25 percentile (EVR, 0.9 vs. CNI, 0.8%; *p* = 0.846) and in BMI category of >25–≤50 percentiles (2.6 vs. 1.1%, *p* = 0.271). However, wound healing complications were significantly higher in >50–≤75 categories (2.0 vs. 0.6%, *p* = 0.049). Majorities of the earlier de novo studies on SRL showed a positive association between wound healing complications and lymphoceles [1, 5, 7, 12, 108–111, 113, 115, 116, 118–125]. In contrary to most studies on EVL, which kept trough level 3–8 ng/mL [10, 14, 69, 131–133, 135, 138, 139], we did not find significance on wound healing or lymphocele formations. These differences could be due to a shorter half-life or higher bioavailability of EVL, or it could be due to loading doses and a very high trough level of 10–30 ng/mL used in the case of SRL. Avoidance of loading dose [116, 127] and use of low-dose SRL have been shown to reduce wound healing complications [115, 127]. No randomized control trial has made a head-to-head comparison between SRL and EVL. An open label RCT is going at the moment, which will compare three arms (EVL-TAC, SRL-TAC, and MMF-TAC). The study will be completed by the end of 2021 and will look into safety profile including wound healing complications between SRL and EVR [141].

The thought that delayed administration of mTOR-I may reduce wound complications and delayed graft function was evaluated in a few RCTs. Albano et al. [132] were the first to assess this strategy in 2009. They compared immediate EVL from day 1 with delayed EVL from week five and found no difference in delayed graft function and wound healing complications. Similarly, the CALLISTO study [10] did not find the difference in wound healing complications and delayed graft functions between immediate or delayed use of EVL. These findings were reinforced in 2020 by Manzia et al. [18], who also found no difference in wound healing complications between immediate or delayed use of EVL.

A couple of the recent studies on mTOR-I further increased the insight into using these agents in de novo transplantation. Schäffer et al. [28] in 2018 used ER-TAC with SRL and compared it with ER-TAC and MMF. This group kept trough level 3–5 ng/mL. Wound healing and risk of lymphocele were not significantly different between the two groups. In the TRANSFORM study, the use of EVL aiming for a trough concentration of 3–8 ng/ml avoided the increased rates of lymphocele though the wound healing complication were slightly higher [53]. Later on, an in-depth analysis of TRANSFORM data was performed by Tedesco et al. They compared wound healing complications with mean EVL concentration during the periods from day 4 to week 4, day 4 to month 2, and day 4 to month 12. They found no significant association of the mean concentration of EVL with wound healing complications [69]. The ATHENA randomized control trial was published in 2019 and compared three arms consisting of EVR/TAC, EVR/CsA, and MPA/TAC and found no difference in wound healing complications among the three groups [139]. NEVERWOUND study was another RCT published in 2020 which compared immediate use of EVL-CsA-Pred with delayed use and found no difference in wound healing complications and lymphoceles between the two arms [18].

## 6. Emergency or Elective Surgery in Patients on mTOR-I

Clear guidelines for continuing mTOR-I in the wake of any emergency or elective surgery after kidney transplantation are lacking. This is simply due to the lack of randomized control trials. Most of the data available are case reports, retrospective studies, or prospective case series. SRL and obesity have been risking factors for hernia recurrence in liver transplant patients [142]. Different approaches have been reported in the literature for patients undergoing surgery. Scheuerlein et al. switched SRL to calcineurin inhibitors in patients undergoing laparoscopic incisional hernia repair after solid-organ transplantation [143]. On the other side, immunosuppression, including mTOR-I, was maintained postoperatively in patients undergoing laparoscopic incisional hernia repair after solid-organ transplantation and aortic valve replacement in kidney transplant patients [144, 145]. Hebel et al., in their retrospective analysis of 13 pediatric cardiac patients who underwent surgery, found that only 1/13 (7.7%) has wound complications [146]. Schwarz et al. studied six liver transplant recipients who underwent nine major abdominal or thoracic surgical procedures without mTOR-I discontinuation or specific dosage adjustment. They found no evisceration, incisional surgical site infection, or lymphocele [147]. However, one has to bear in mind that patients in this retrospective analysis did not include obese patients and the overall mean SRL trough concentration was 4.8 ng/mL. Campistol et al. made recommendations for minor surgery, major surgery, and emergency surgery in patients on SRL [148]. They kept into consideration nonmodifiable risk factors (age, African American) and modifiable risk factors (obesity >26 kg/m^2^, use of steroids, and use of ATG and anticoagulant) while deciding mTOR-I in the event of surgery. They suggested that no change is required in minor surgery or laparoscopic surgery without risk factors. In major surgery, including those who required chemotherapy, the group recommended holding SRL 5–10 days before the operation and restarting 1–3 months later. They suggested stopping mTOR-I immediately and restarting five days later in emergency surgery.

In the absence of robust data, it is challenging to advise about the withdrawal of mTOR-I in the wake of surgery. While planning for elective surgeries, it is crucial to look for risk factors of wound healing and reduce the dose of mTOR-I to ensure lesser chances of wound healing complications and prevent rejection at the same time. In emergency surgery in patients with risk factors for wound healing or postoperative wound complications, a decision of withdrawal may be considered.

## 7. Way Forward for the Use of mTOR-I

Minimization of CNI while using mTOR inhibitor provides synergistic immunosuppressive effects and reduces nephrotoxicity. mTOR-I has an antiviral and antitumor effect [4, 6]. The incidence of cytomegalovirus and BK virus infections in EVL is significantly lower when used with minimized CNI and steroids compared to the combination of MMF [53]. Therefore, it is important to use these agents wisely to achieve their maximum potential benefits and to keep its side effects minimum possible level.

**Figure 2:**
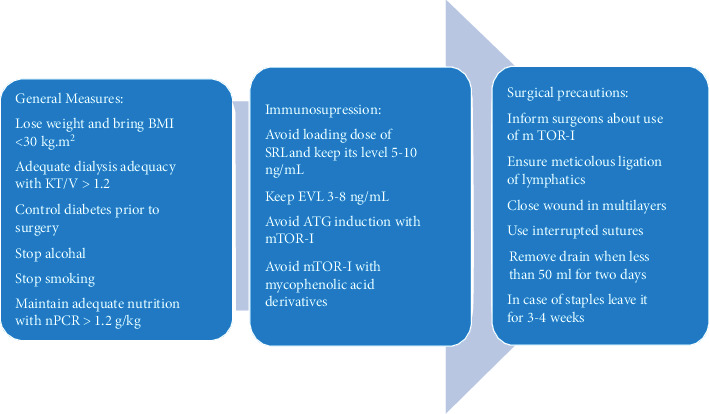
Showing the way forward to minimize wound healing.

Since the introduction of SRL in 1972 and multiple randomized control trials on EVL since the start of 2000 with the availability of significant amount of evidence, much has been known about these agents. Events of cadaveric kidney transplantation are not planned events and clinicians do not have enough time to optimize nonimmunological risk factors for wound healing complications. In contrast to cadaveric transplantation, live kidney transplantation gives clinicians a chance to optimize the kidney transplant recipients before surgery to ensure a better outcome. Therefore, live kidney transplant recipients should be optimized before the planned surgery. Nonimmunological risk factors should be identified and discussed with the recipients to avoid wound healing complications and optimize recipient and graft survival. Those selected to be a candidate for kidney transplantation should be thoroughly evaluated for wound healing risks. Those with BMI greater than 30 kg/m^2^ should be encouraged to lose weight. BMI in lower ranges has been significantly associated with reduced wound healing complications [64]. Detailed smoking history should be obtained, and it is essential to stop smoking six weeks before kidney transplantation [95]. Similarly, alcoholics should be encouraged to quit drinking to reduce wound healing complications. Potential live recipients with a history of diabetes should have optimal diabetes control before transplant to minimize the perceived complications [84]. Patients undergoing renal replacement therapy must have adequate dialysis in the preceding months before transplantation to minimize the effect of uremia on wound healing complications [75]. Clinical evaluation should be performed along with an estimation of protein catabolic rate to identify malnourished patients. These patients should be treated with dietitians to improve their nutritional status [103, 104]. These patients must have nPCR between 1.0 and 1.2 g/kg/d. The serum albumin should be equal to or greater than 4.0 g/dL [104].  Figure 2 shows the way forward to minimize wound healing complications.

Previous higher wound healing complications were attributed to higher loading doses of SRL (ranging from 6 mg to 30 mg) along with higher trough level of 10–30 ng/mL [1, 5, 7, 12, 108–111, 113, 115, 116, 118–125]. Avoidance of loading has been shown to reduce wound healing complications significantly [116]. Use of low-dose SRL has been shown to reduce incidence of lymphocele significantly when compared with higher dose [11]. Therefore, we suggest to avoid loading dose and keep trough level between 5–10 ng/mL [149, 150] to minimize adverse events and wound healing complications. If one contemplates using SRL with TAC-ER, SRL level can be kept even low at 3–5 ng/mL [127]. Low-dose EVL when compared with high-dose EVL (1.5 mg/day vs. 3 mg/day) led to numerically a smaller number of wound healing complication [2, 3, 8]. Higher blood level of EVL (>8 ng/mL) has been shown with increased risk (HR, 1.69; 95% CI, 1.20–2.38; *p* = 0.002) [134]. Therefore, we suggest EVL level to be kept between 3–8 ng/mL [10, 14, 69, 131–133, 135, 138, 139]. ATG induction in patients with mTOR-I should be avoided to reduce wound healing complications [18, 62, 63]. Since most candidates for mTOR-I are of low immunological risk, induction with basiliximab will be a reasonable option. The combination of SRL with MMF has synergetic effects on wound healing, as reported by the SYMPHONY trial. Therefore, mTOR-I, especially SRL, should be avoided with MMF or MPA [1]. Steroid's dose should be minimized to reduce the risk of wound healing [151].

Planning surgery also plays a vital role in preventing wound healing complications. Surgeons must be aware of the potential use of mTOR-I in the posttransplant period. Tiong et al. analyzed a systemic approach to minimize wound healing complications in de novo SRL [140]. Their approach included patient selection (body mass index) [BMI] < 32 kg/m^2^, the use of closed suction drains, modifications of surgical technique, and avoidance of a loading dose of SRL. Surgical wound closure was performed via a multilayer closure approach using nonabsorbable interrupted sutures in the fascia. The skin closure was performed through interrupted nonabsorbable monofilament sutures. The drains were removed after 2–3 weeks or when drain volume was less than 50 ml for two days. The sutures were usually left for three weeks in the majority of patients. Using this approach, a significant reduction was found in cumulative wound complications (7.8% vs. 19.6%, *p* < 0.007) and lymphocele (22.3% vs. 47.1%, *p* < 0.0001) as compared to the historical cohort [140], leaving staples for 3–4 weeks and draining till drainage minimizes wound dehiscence and collection [148]. Ligation of lymphatic meticulously, peritoneal fenestration, and minimizing dissection will reduce lymphocele formation [151].

## 8. Conclusion

mTOR-I can be used immediately after kidney transplantation. Loading doses and high trough levels for SRL lead to more wound healing complications and should be avoided. EVL trough level of 3–8 ng/mL maintains its efficacy and avoids most adverse events, including wound healing complications. Induction with ATG may be avoided. mTOR-I should be used with low-dose CNI, and its combination with mycophenolic acid derivatives should be avoided. Patients with BMI ≥30 kg/m^2^ should be encouraged to lose weight before surgery. Adequate nutrition, cessation of smoking and alcoholism, controlling diabetes, and adequate dialysis before transplant surgery can minimize wound healing complications. Surgical wound closure in multilayers using interrupted suture, meticulous ligation of lymphatics, leaving staples for 3–4 weeks, and using close suction drain decrease wound healing complications [152] (Table 4).

## Figures and Tables

**Table 1:** Animal studies on mTOR-I to study its impact on wound healing.

Reference	Journal/year	Objective	Intervention	Finding
Dantal et al. [10]	Faseb Journal/2002	Hypoxia increases DNA synthesis and proliferative response to platelet-derived growth factor (PDGF) and ﬁbroblast growth factor (FGF) in rat and human smooth muscle and endothelial cells. It is dependent on mTOR activation downstream enzyme phosphatidyl inositol 3-kinase. Rapamycin blocks these effects and inhibits fibrogenesis and angiogenesis.	Primary cultures of rat aortic smooth muscle cells were isolated from fresh rat aortas. Aortas were denuded from endothelium and adventitia and the aortic media was fragmented mechanically and subcultured. Effect of hypoxia and activity of PI3K were analyzed. Effect of rapamycin on hypoxia induced proliferation was also analyzed.	Hypoxia increases PDGF- and FGF-induced proliferation of vascular wall cells. PI3K activity is required for the proliferative response of vascular cells and angiogenesis in vitro under normoxia and hypoxia. Rapamycin speciﬁcally inhibits the hypoxia-mediated increase in growth factor-mediated vascular cell proliferation.
Vitko et al. [11]	Journal of Immunology/2008	Intraepithelial lymphocytes, *γδ*T cells in the skin helps in tissue repair via cytokine and growth factor. The function of this T cell was analyzed as rapamycin treatment in a mouse model of wound repair.	Wildtype C57BL/6J mice were given daily rapamycin or vehicle control for three days before wounding. This treatment was continued after wound for total of 14 days.	Fewer wounds were closed on day 10 in rapamycin-treated mice as compared to vehicle control treated animals. Delay on day 3 wound closure was found which was similar mice lacking *γδ*T cells (TCR*δ*−/− mice).
Durrbach et al. [12]	Transplantation/2006	To study the effect of EVL on wound healing in rat intestine.	4 groups of male Wistar rats were given 0 mg (group 1, control), 0.5 mg (group 2), 1 mg (group 3), and 3 mg (group 4) starting 4 hours before colonic and iliac anastomosis until killing on day 3 or day 7 of operation.	There was no difference on day 3. Breaking strength and bursting pressure were reduced on day 7 in EVL group. There was reduction in hydroxyproline content and there was less collagen deposition in the wound. The effects were more pronounced in higher EVL group of 3 mg.
Vitko et al. [13]	Wound repair and regeneration/2009	To study the effect of EVL in both intestine and abdominal wall in rats over a period of 4 weeks.	Wistar rats received a daily dose of 1 or 2 mg/kg EVL orally, from the operation day and control was given saline onwards.. Controls received saline. All rats have resection of ileum and colon with end-to-end anastomosis, and on day 7, 14, and 28, animals were killed. Their abdominal and anastomotic wounds were assessed for wound strength.	Wistar rats received a daily dose of 1 or 2 mg/kg EVL orally, from the operation day onwards. Controls received saline. In each rat, a resection of ileum and colon was performed, and end-to-end anastomoses were constructed. On day 7, 14, and 28, the animals were killed and anastomoses and abdominal wall wounds were analyzed, wound strength being the primary parameter. Breaking strength of ileum, colon, and fascia was consistently and signiﬁcantly reduced in the experimental groups. Anastomotic bursting pressures followed the same pattern. Loss of strength was accompanied by a decrease in hydroxyproline content after 7 days. Thus, the negative effect of EVL on wound repair persists for at least 4 weeks after operation in this rodent model. This protracted effect may have clinical consequences and cause surgical morbidity.
Salvadori et al. [14]	Transplantation Proceeding/2007	To see the effects of rapamycin on the healing of bladder and abdominal wound closures.	Study was done in 14 male rats. Rapamycin (3 mg/d) or placebo was given to them. Midline abdominal incision was given and bladder was cut and closed with 4–0 vicryl.	Eosinophil and neutrophil infiltration and myofibroblast proliferation were significantly higher in bladder, fascia, and dermis of the control group. Lymphocyte's infiltration was the same in both groups. Mean microvessel density as well as the percentage of cells expressing vascular endothelial growth factor in the bladder, fascia, and dermis were significantly lower among rapamycin.
Büchler et al. [15]	Transplantation Proceeding/2005	To see the effect of rapamycin on wound healing and the healing of the ureteric anastomosis.	Pigs underwent laparotomy and excision of the ureter followed by anastomosis of the ureter. The animals were randomly allocated to receive either rapamycin or placebo. The animals were sacrificed on postoperative day 5. Skin, fascia, and ureteric tissues were assessed for the tensile strength, hydroxyproline levels, and histological changes.	The tensile strength and the hydroxyproline levels in the ureter and fascia were lower in the rapamycin-treated group. However, there was no difference in the tensile strength in the skin, although the hydroxyproline levels were lower.
Flechner et al. [16]	European Journal of Cardiothoracic Surgery/2003	SDZ RAD (40–0 (2-hydroxyethyl)-rapamycin), a rapamycin derivative, inhibits fibroblast proliferation and may limit development of bronchiolitis obliterans. But, it may impair the healing of the bronchial anastomoses.	The cervical trachea in pigs was divided and reanastomosis was done. One group was given SDZ RAD for 14 days and control group was given none.	SDZ RAD significantly reduced the breaking strength of the tracheal anastomosis. However, no differences in histological samples were found between two groups.

**Table 2:** Experimental and clinical studies performed on mycophenolic acid derivatives and their effects on wound healings.

Reference	Journal/year	Objective	Intervention	Finding relevant to fibroblast growth and wound healing
Pilmore et al. [2]	American Journal of Transplantation/2008	To show the effects of MPA on human fibroblast proliferation, migration, and adhesion in vitro and in vivo and its implication on wound healing	Human fibroblast was cultured. Expression of cytoskeletal proteins vinculin, actin, and tubulin in fibroblasts was assessed by polymerase chain reaction (PCR) and western blot. RNA and protein content and its effect on rearrangement on cytoskeleton was assessed by immunofluorescence. Scratch test was done to assess reduced migration activity. The results of the cultured human fibroblasts were applied to skin biopsies of renal transplant recipients. Skin biopsies of patients treated with MPA were assessed with control.	The authors showed a downregulation of the cytoskeletal proteins vinculin, actin, and tubulin in fibroblasts exposed to pharmacological doses of MPA. This reduction in RNA and protein content is accompanied by a substantial rearrangement of the cytoskeleton in MPA-treated fibroblasts. The dysfunctional fibroblast growth was validated by scratch test documenting impaired migrational capacity. In contrast, cell adhesion was increased in MPA-treated fibroblasts. The results of the cultured human fibroblasts were applied to skin biopsies of renal transplant recipients. Skin biopsies of patients treated with MPA expressed less vinculin, actin, and tubulin as compared to control biopsies.
Engels et al. [3]	Transplant Direct/2016	To study the effect of MMF on wound healing in rodent model	4 groups were made from ninety-six male Wistar rats. All the groups underwent anastomotic construction in ileum and colon at day 0. Three groups received daily oral doses of 20 or 40 mg/kg MMF or saline (control group) from day 0 until the end of the experiment. Half of each group was analyzed after 3 days and half after 7 days. 4th group started the medication 3 days after the laparotomy and was analyzed after 7 days. Half of the 4th group received 20 mg/kg and half 40 mg/kg MMF. Wound strength in anastomoses and in the abdominal wall was measured by assessing bursting pressure, breaking strength, and histology.	On day 3, it was shown that there was stronger anastomosis in the experimental groups. Bursting pressure as well as breaking strength were higher in the low-dose and high-dose MMF group compared with the control group. However, wound strength in abdominal wound was less in the highest MMF group.
Yanik et al. [4]	Int Braz J Urol/2014	To study the synthesis of type I (mature) and type III (immature) collagen in bladder suture of rats treated with a combination of TAC and MMF for 15 days.	Thirty rats were grouped into 3 groups: the sham (did not receive any treatment), control (saline solution), and experimental groups (received 0.1 mg/kg/day of TAC with 20 mg/kg/day of MMF). All treatments were given for 15 days. All the animals underwent laparotomy, cystotomy, and bladder suture in two planes with surgical PDS 5–0 thread. The surgical specimens of the bladder suture area were assessed for the type of collagen deposition.	Type I collagen production and deposition in the sham and control groups were more as compared to the experimental group. TAC and MMF change qualitatively to collagen type III in wound
Eckl et al. [5]	Br J Ophthalmol/2003	To study if growth inhibition of MMF on human tenon fibroblasts is mediated by guanosine depletion.	Human tenon fibroblasts were cultured incubated in various concentrations of MMF with and without supplementation of guanosine.	Tenon fibroblast growth was inhibited in a concentration-dependent way. It was reversed by guanosine supplement.
Tedesco-Silva et al. [6]	Nephrol Dial Transplant. 2000 Feb; 15 (2):184–90.	To study the effect of MMF on proximal convoluted tubules (PCT) and distal convoluted tubules (DCT).	Human PTC and DTC were cultured in the presence of different concentrations of MPA (0.25–50 microM) or MPA plus guanosine (100 microM). Cells were stimulated by a combination of cytokines. Secretion of RANTES protein was evaluated. Cell surface expression of HLA-DR and ICAM-1.	MPA inhibited cell growth of PTC and DTC in a dose-dependent manner. This effect was totally abolished by the addition of guanosine.
Franz et al. [7]	Kidney International/2002	To study effects of MPA human mesangial cells (HMC) activation.	Primary cultures of HMC and of an immortalized HMC clone were stimulated and treated by MPA at clinically relevant concentrations (1 to 10 mol/L) for 24 hours.	Treatment of cultured HMC with MPA inhibited mesangial cell proliferation and matrix production.
Dean et al. [8]	Nephrol Dial Transplant/1999	To study the effect of mesangial cell (MC) proliferation in inflammatory proliferative glomerular diseases.	The growth of fetal rat and human MCs were arrested by taking out fetal calf serum (FCS) and then stimulated by addition of FCS, platelet-derived growth factor (PDGF), or lysophosphatidic acid. Different concentrations of MMF (0.019–10 microM) were added concomitantly in the presence or absence of guanosine.	MMF inhibited mitogen-induced human rat MC proliferation. The effect on human MC was more pronounced.
de Fijter et al. [9]	Kidney Int/2000	To investigate the effect of MMF on whether it reduces interstitial myoﬁbroblast inﬁltration.	Forty-five rats underwent renal ablation. One group received daily dose of vehicle (N 5/20). The other group received MMF (N 5/25). This was continued during the 60 days following surgery.	Cellular proliferation in renal tubules, interestitium, and glomeruli along with myoﬁbroblast inﬁltration in interestitium and interstitial type III collagen deposition were signiﬁcantly reduced by MMF treatment. MPA showed a dose-dependent inhibitory effect on in vitro proliferation of rat ﬁbroblasts. MMF treatment improved renal function and resulted in reduced kidney hypertrophy and glomerular volume parameters and progressively decreased remnant kidney hypertrophy and glomerular volume increment.
Dantal et al. [10]	Transplantation/1998	The objective of the study was to avoid the nephrotoxic effects by CsA avoidance using MMF during induction and maintenance.	In primary CsA, free induction methyl prednisolone and ATG were given during induction and oral MMF 1 gram twice a day was given within 24 hours after surgery. In late group, CsA was withdrawn after 4 weeks slowly (25 mg/day) and was kept on MMF.	Wound healing complications occurred in 16.6% of MMF treated patients.
Vitko et al. [11]	Kidney Blood Press Res/2010	Retrospective study to assess MMF on wound healing and lymphocele formation.	Retrospective single-center analysis of 144 patients receiving a CsA-based immunosuppression with prednisolone (Pred) and either MMF (*n* = 77) or AZA (AZA, *n* = 77) was done. The end points were lymphocele and nonprimary wound healing during 6 months follow-up.	More lymphoceles were observed in MMF group (OR = 2.6; *p* = 0.03). More fluid drainage (17 vs. 5 interventions) and sclerotherapies (8 vs. 0) were done in MMF group.
Durrbach et al. [12]	Am J Transplant/2003	Retrospective analysis of effect of SRL vs. MMF on surgical complications and wound healing in adult kidney transplant recipients.	Patients on MMF and SRL were retrospectively analyzed for wound healing complication via logistic regression analysis.	The incidence of wound complications was statistically different for patients receiving MMF compared to SRL: 2.4% for group 1 vs. 43.2% for group 2 (*p* < 0.0001).
Vitko et al. [13]	Am J Transplant/2007	This prospective randomized study was done to compare the safety and efﬁcacy of an SRL-MMF-based regimen with a CsA -MMF-based regimen after induction therapy with polyclonal antilymphocyte antibodies, with withdrawal of steroids 6 months.	Primary end point was graft function at 12 months. Secondary outcome included acute rejection, delayed graft function, slow graft function, and CMV infection.	Hernial eventration/wound evisceration was in 7/71 in SRL-MMF group as compared to 0/71 in CsA-MMF group
Salvadori et al. [14]	Am J Transplant/2011	To assess safety and efﬁcacy of two SRL, dosing regimens were compared with TAC and MMF (ORION study)	Patients were randomized to group 1 (SRL + TAC); week 13 TAC elimination, group 2 (SRL + MMF), or group 3 (TAC + MMF)).	Delayed wound healing was present in 16.4% (SRL-TAC elimination group) and 23% (SRL + MMF) with *p* < 0.05 between the two groups. Lymphocele was present in 18.4% in SRL-MMF group.
Büchler et al. [15]	J Am Soc Nephrol/2018	To assess adverse events in kidney transplant recipient who received different immunosuppressive	Kidney transplant recipient undergoing kidney transplant received low-dose SRL or CsA (TAC or SRL) in addition to daclizumab induction or standard-dose CsA without induction. All patients received MMF and corticosteroids.	17% patients have delayed wound healing in low-dose SRL and MMF and was significant as compared to another group with *p* value = 0.006. The incidence of lymphocele was 15.8% in low-dose SRL-MMF and was significant as compared to other groups with *p* value < 0.001.
Pengel LH et al. [SRL 16]	Transpl Int/2011	To do metanalysis to assess if wound complications or lymphoceles occur more often in solid-organ transplant recipients on mTOR inhibitors.	Metanalysis of 17 randomized control trials was done.	Incidence of wound healing complications (OR 3.00, CI 1.61–5.59) and lymphocele (OR 2.13, CI 1.57–2.90) were significantly higher in mTOR-I and MMF as compared to mTOR-I and calcineurin inhibitor where incidence of wound healing complications was (OR 1.77, CI 1.31–2.37) and that of lymphoceles (OR 2.07, CI 1.62–2.65) [17].

**Table 3:** Summaries of randomized control trial performed on SRL.

Reference	Journal/year/design	Induction therapy	Arms compared	Loading dose used (yes/no)	SRL dose and level	Finding
Groth et al. [108]	Transplantation/1999/open label study	Methyl prednisolone	1-SRL-AZA-Pred 2-CsA-AZA-Pred	Yes (loading dose 16–24 mg/m^2^/day for day 1, then 8–12 mg/m^2^/day for day 7–10)	Trough level 30 ng/mL for first two months and 15 ng/mL thereafter	Wound infection in 10% of SRL-aza-Pred as compared to 5% CsA-aza-Pred
Kreis et al. [109]	Transplantation/2000/open label study	-----------------------------	1-SRL-MMF-Pred 2-CsA-MMF-Pred	Yes (loading dose: 24 mg/m^2^ for three days)	Trough levels were kept at 30 ng/mL for 2 months and 15 ng/mL afterward	Wound infection in 5% of SRL-MMF-Pred as compared to 8% in CsA-MMF-Pred
Kahan [110]	Lancet/2000/		1-SRL-CsA-Pred (low dose) 2-srl-CsA-Pred (high dose) 3-aza-CsA-Pred	Yes (6 mg loading dose followed by 2 mg per day in low dose and 6 mg per day followed by 5 mg in high-dose group)	-------------------------------------	Lymphocele occurred in 12% low-dose SRL, 15% high-dose SRL, and 3% in AZA group *p*<0.001 for SRL 5 mg/d vs. AZA
Flechner et al. [111]	Transplantation/2002/open label study	Basiliximab 2 doses	1-SRL-MMF-Pred 2-CsA-MMF-Pred	Yes (loading dose 15 mg)	Trough 10–12 ng/mL for 6 months and 5–10 ng/mL afterward	Wound infection happened in 6.5% of SRL-MMF-Pred as compared to 3.3% in CsA-MMF-Pred lymphocele was reported in 9.7% of SRL-MMF-Pred and 3.3% of CsA-MMF-Pred
Dean et al. [8]	Transplantation/2004/open label study	ATG	1-SRL-MMF-Pred 2-TAC-MMF-Pred	Yes (loading dose 10 mg/d for 2 days)	Trough level 15–20 ng/mL for 4 months and 10–15 ng/mL thereafter; reduced to 10–15 ng/mL throughout after recruitment of first 77 patients	Wound healing complication was found in 47% in SRL-mm-Pred as compared to 8% in TAC-MMF-Pred
Machado et al. [113]	Clin Transplant/2004/open label study	---------------------------------	1-SRL-reduced CsA-Pred 2-aza-CsA-Pred	Yes (loading 6 mg was given)	Maintain at 2 mg/day	Wound related complications happened in 40% of SRL-reduced CsA-Pred as compared to 20% of AZA-CsA-Predp = 0.117S
Lo et al. [114]	Transplantation/2004	ATG and corticosteroids administered in both groups	1-TAC sparing regimen: standard SRL + reduced TAC 2-TAC-free regimen: full-dose SRL + MMF	Yes (SRL 10 mg loading dose was given after 48 hours for 2 days)	SRL (C0, 10–15 ng/mL) + reduced SRL full-dose (C0, 12–15 ng/mL)	Wound complication occurred in 17% of the subjects in the TAC sparing group and in 7% of the subjects in the TAC-free group (*p*=NS). The incidence of lymphocele or fluid collections was 20% in the CI-sparing and 10% in the CI-free group (*p*=NS)
Ciancio et al. [115]	Transplantation/2004/open label study	Daclizumab and methyl prednisolone	1-SRL-TAC-Pred 2-SRL-CsA-Pred 3-TAC-MMF-Pred	Yes (A SRL loading dose of 4 mg was given to both groups on SRL on evening of the surgery followed by maintenance)	*C*0 = 8 ng/mL C0 = 8 ng/ml	Wound complication 6%, lymphocele 16% Wound complication 4%, lymphocele 14% Wound complication 4%, lymphocele 6% *p* = 0.27
Kandaswamy et al. [116]	Am J transplant/2005/open label	ATG is followed by	1-CsA-MMF 2-high-dose TAC, low-dose SRLv3-low-dose TAC-high-dose SRL	Loading dose was given before December 1, 2002 No loading dose was given after December 1, 2002	*C*0 = 7 ng/mlc0 = 8–12 ng/mL	Wound healing complications were 8%, 18%, and 25% in CsA-MMF, low-dose SRL, and high-dose SRL, *P* = 0.02 Wound healing complications reduced to 5%, 11%, and 20% in CsA-MMF, low-dose SRL, and high-dose SRL, *P* = 0.3 after stopping loading dose after December 1, 2002
Hamdy et al. [117]	Am J Trans /2005/open label study	Basiliximab	1-SRL (standard dose)-TAC-Pred 2-srl (full dose)-MMF-Pred	Yes Group 1: full dose of 10 mg/day orally (single morning dose) for 3 days and then maintained at 5 mg/day Group 2: a single oral morning dose of 10 mg/day	SRL (C0, 6–12 ng/mL) SRL (C0, 10–15 ng/mL)	There were 7 wound complications, 4 lymphoceles, and 1 urinary fistula in TAC sparing regimen. There were 11 wound complications, 7 lymphoceles, and 2 urinary fistulas in TAC-free regimen. Surgical complications between two groups were not significant (*p* = 0.127)
Larson et al. [118]	Am J Transplant/2006/open label study	ATG/methyl prednisolone	1-SRL-MMF-Pred 2-TaC-MMF-Pred	Yes (SRL was initially dosed at 10 mg daily for 2 d and 5 mg daily thereafter)	SRL C0 were kept 15–20 ng/mL in the first 4 months and 10–15 ng/mL thereafter	18 patients in SRL group discontinued SRL because of wound healing complications
Vitko et al. [11]	Am J Transplant/2006/open label study	No induction therapy	1-SRL-TAC-Pred 2-SRL-TAC-Pred 3-MMF-tacrolmus-Pred	Yes 1-SRL-TAC-Pred: loading dose 1.5 mg, then maintenance 0.5 mg per day 2-SRL-TAC-Pred loading dose 6 mg, then 2 mg per day	Median C0 levels in the TAC‐SRL0.5 mg group were 0.95 ng/mL during week 1, 1.46 ng/mL at month 3, and 1.43 ng/mL from 4 to 6 months Median C0 level TAC-SRL 2 mg group was 2.71 ng/mL during week 1, 4.57 ng/mL at month 3, and 4.75 ng/mL between months 4 and 6	Low SRL group has lymphocele in 4.3% as compared to 8.6% in high SRL group. *p* = 0.022
Martinez-Mier et al. [119]	Transplantation/2006/open label RCT study	Basiliximab	1-SRL-MMF-Pred 2-CsA-MMF-Pred	Yes (10 mg loading dose followed by 3 mg/m body surface area/day)	24 hr trough levels were kept at 10–15 ng/mL for six months and 5–10 ng/mL afterward	Wound infection occurred in 15% in SRL-MMF-Pred group as compared to 9.5%
Ekberg et al. [1]	N Eng J Med/2007/open label RCT study	Daclizumab	1-standard-dose CsA-MMF-Pred 2-low-dose CsA-MMF- Pred 3-low-dose TAC-MMF-Pred 4-low-dose SRL	Yes (oral SRL at a dose of 9 mg per day for 3 days and 3 mg per day to aim for target level *C*0 = 4 to 8 ng/mLl)	*C*0 = 4 to 8 ng/mL	The incidence of lymphocele was double in low-dose SRL as compared to other groups which was high (*p* < 0.001). The incidence of delayed wound healing was also significantly high in low-dose SRL as compared to other *p* = 0.006
Büchler et al. [15]	Am J Transplant/2007/open label RCT study	Antilymphocyte antibodies	1-SRL-MMF-Pred 2-CsA-MMF-Pred	Yes (loading dose 15 mg for 2 days, then 10 mg for 1 day)	*C*0 = 10–15 ng/mL	-Hernia/evisceration occurred in 9.9% vs. 0% in SRL group and CsA group comparatively (*p*=0.006) Lymphocele occurred in 11.3 and 5.4%, respectively, between two groups but were not significant
Pescovitz et al. [120]	Br J Clin Pharmacol/2007/open label RCT study	Daclizumab	1-SRL-MMF-Pred 2-CsA-MMF-Pred	No	*C*0 = 10–25 ng/*m* L for first 2 months posttransplant, and 8–15 ng/mL afterward	Incision site complications were 40% and 20%, respectively
Gaber et al. [121]	Transplantation/2008/open label RCT study		1-SRL-TAC-Pred 2-SRL-CsA-Pred	Yes (SRL 10 mg loading dose for first two days and then 5 mg once daily were given in both groups)	*C*0 = 10–25 ng/*m* L	Delayed wound healing between two groups (SRL-TAC-Pred 13.4% vs. SRL-CsA-Pred 16.5%) was not significant Lymphocele occurred significantly more in SRL-CsA-Pred (27.2% vs. 18.8%) in SRL-TAC-Pred group (P0.043)
Durrbach et al. [12]	Transplantation/2008/open label RCT study	ATG	1-SRL-MMF-Pred 2-CsA-MMF-Pred	Yes (loading dose of 30 mg for two days)	*C*0 = 10–20 ng/m L	Lymphocele occurred in 24.2% vs. 2% of the cases between the two groups, *p* = 0.04%
Sampaio et al. [122]	Clin Transplant/2008/open label RCT study	No induction	1-SRL-TAC-Pred 2-MMF-TAC-Pred	Yes (loading dose 15 mg, then 5 mg per day for 7 days and then 2 mg per day continued)	-------------------	Wound healing complications occurred in 34% in SRL group as compared to 10% in MMF group (*p* = 0.007)
Franz et al. [7]	Am J Kidney Dis/2010/open label RCT study	--------------------	1-SRL-MMF-Pred 2-CsA-MMF-Pred	Yes (30 mg for 3 doses)	*C*0 = 10–20 ng/mL for 3 months and then 8–15 ng/mL afterward	Impaired wound healing occurred in 3.2% of SRL group as compared to 1.6% in CsA group Lymphocele occurred in 14.3% of SRL group as compared to 3.1% in CsA group *p* = 0.03
Glotz et al. [123]	Transplantation/2010/open label RCT study	ATG	1-SRL-MMF-Pred 2-TAC-MMF-prd	Yes (15 mg for two days and then 10 mg for 5 days)	*C*0 = 10–20 ng/*m* L	Impaired wound healing occurred in 11.3% vs. 0 % *p* = 0.006
Ekberg et al. [124]	Nephrol Dial Transplant/2010/open label RCT study	Daclizumab	1-standard-dose CsA-MMF-Pred 2-low-dose CsA-MMF-Pred 3-low-dose TAC-MMF-Pred 4-low-dose SRL	No	*C*0 = 4 to 8 ng/mL	Wound healing complications occurred in 11%, 11%, 9%, and 17%, respectively, in each group and was significantly higher across the group (*p*=0.006) Lymphocele occurred in 7%, 6.8%, 9%, and 15.8%, respectively and *p*= <0.001 across groups for lymphocele formation
Flechner [16]	Am J Transplant/2011/open label RCT study	Daclizumab	1-SRL-TAC for 3 months-Pred 2-srl-MMF-Pred 2-MMF-TAC-Pred	Yes (loading dose of 15 mg given to both SRL groups)	*C*0 = 8–15 ng/mL for 3 months and then 10–20 ng/mL afterward in SRL-TAC-MMF group C0 = 10–15 ng/mL for 3 months, then 8–15 ng/mL for 3–6 months and then 5–15 ng/mL afterward	Delayed wound healing occurred in 16.4% in SRL-TAC-Pred, 23% in SRL-MMF-Pred, and 5.8% in MMF-TAC-Pred. *p* < 0.01 for delayed wound healing for SRL-TAC-Pred as compared to MMF-TAC-Pred Lymphocele occurred in 16.4% in SRL-TAC-Pred, 18.4% in SRL-MMF-Pred, and 8.6% MMF-TAC-Pred
de sandes freitas et al. [125]	Int Urol Nephrol/2011/open label RCT study	No induction	1-TAC-SRL 2-SRL-Pred	-----------------	-------------------	Higher incidence of lymphocele or lymphorrhea was observed in TAC-SRL group as compared to SRL-Pred (13 vs. 4.1%)
Flechner [126]	Transplantation/2013/open label RCT study	IL-2 receptor antagonist	1-SRL-MMF-Pred 2-CsA-MMF-Pred	Yes (initially 10 to 15 mg SRL oral loading dose within 2 days, 4 to 8 mg daily) Amendment was done and SRL group received two 15 mg oral loading doses within for 2 days followed by 10 mg daily to achieve *C*0 = 10.0 ng/mL or more	*C*0 = day 1 to week 13, 10–15 ng/mL; weeks 14–26, 8–12 ng/mL; weeks 27–104, 5–12 ng/mL. After amendment *C*0 = day 1 to week 26, 10–15 ng/mL; weeks 27–104, 8–15 ng/mL.	15.2% of SRL and 8.2% of CSA had wound healing complication, *p* = 0.033
Huh et al. [127]	Nephrol Dial Transplant/2017/open label RCT study	Basiliximab	1-ER-TAC-MMF 2-ER-TAC-SRL	No	2 mg of SRL was given within 24 hours of transplant, C0 = 3–5 ng/mL.	SRL group has 10.5% wound healing complications as compared to 2.7% in MMF group, but *P* value = 0.10 was not significant Risk of lymphocele was 0% in SRL group and 1.3% in MMF (*P* = 0.10)

**Table 4:** Summaries of randomized control trial performed on EVL.

Reference	Journal/year/design	Induction	Arms compared	EVL dose	EVL level	Finding
Vitko et al. [13]	Am J Transplant/2004/2 open label RCT studies	Study 1: without basiliximab Study 2: with basiliximab	EVL 1.5 mg-CsA-Pred vs. EVL 3 mg-CsA-Pred without basiliximab vs. EVL 1.5 mg-CsA-Pred vs. EVL 3 mg-CsA-P ped with basiliximab	Both arms, two doses of EVL 1.5 mg/day vs. 3 mg/day	>3 ng/*m* L	Study 1: 15.2% had lymphocele in 1.5 mg group as compared to 6.4% in 3 mg group Study 2: lymphocele was found in 10.3% in 1.5 mg EVL group as compared to 7.2% in 3 mg group No comment on *p* value
Vítko et al. [128]	Am J Transplant/2005/double blind RCT study	----------------	1-EVL-CsA-Pred 2- EVL-CsA-Pred 3-MMF-CsA-Pred	1.5 mg/day 3 mg/day	>3 ng/*m* L	Lymphocele occurred in 9% in EVL 1.5 mg-CsA-Pred as compared 12% in EVL 3 mg-CsA-Pred group and 4% in MMF-CsA-Pred (*p* value not significant)
Lorber et al. [129]	Transplantation/2005/open label RCT study	Methyl prednisolone	1-EVL-CsA-Pred 2- EVL-CsA-Pred 3-MMF-CsA-Pred	1.5 mg/day 3mg/day	>3 ng/*m* L	Lymphocele occurred in EVL 1.5 mg group in 16.1%, 18.6% in EVL 3 mg, and 12.2% in MMF-CsA-Pred group. *p* value was insignificant
Chan et al. [130]	Transplantation/2008/open label RCT study	Basiliximab	1-EVL-low-dose TAC-Pred 2-evl-standard-dose TAC-Pred	1.5 mg/day	>3 ng/mL	Wound infection (4.1%), dehiscence (2.1%), and lymphocele (4.1%) occurred in low-dose TAC Wound infection (2.3%), dehiscence (4.7%), and lymphocele (2.3%) occurred in standard-dose TAC. No *P* value given
Margreiter et al. [131]	Transplantation/2008/pool analysis of 4 RCT trial	-------------------	1-MMF-CsA-Pred 2-EVL (1.5 mg/day or 3 mg/day-low-dose CsA-Pred	1.5 mg/day or 3 mg/day	3–8 ng/mL	Wound infection, dehiscence, and lymphocele (4.1% occurred in 9.7%, 3.6%, and 8.4% of MMF group, respectively, in EVL group wound infections were 11.4%, dehiscence was in 6.1%, and lymphocele in 7.5 % similar complication rates. No *P* value given
Albano et al. [132]	Transplantation/2009/open label RCT study	Basiliximab/daclizumab	1-immediate EVL (Day 1)-CsA-Pred 2-delayed EVL (from week 5) MMF was given till week 5 along with CsA-Pred	0.75 mg twice a day adjusted to achieve 3–8 ng/mL	*C*0 = 3–8 ng/mL	Wound healing complication at week 4 was 23.1% vs.29.7% in immediate vs. delayed EVL group (P0.444) Wound healing complication at 3 months was 36.9% vs. 37.8% in immediate vs. delayed EVL group (*P* = 1) Fluid collection at week 4 was 23.1% vs. 25.7% in immediate vs. delayed EVL group
Salvadori et al. [14]	Transplantation/2009/open label randomized control trial	Basiliximab	1-EVL-low-dose CsA-Pred 2- EVL-very low-dose CsA-Pred	0.75 mg twice a day adjusted to achieve 3–8 ng/mL 0.75 mg twice a day adjusted to achieve 3–8 ng/mL during first week and then adjusted to achieve 6–12 ng/mL	*C*0 = 3–8 ng/*m* L C0 = 6–12 ng/mL	Lymphocele in 15.4% Lymphocele in 21.1% The findings were not significant
Silva et al. [133]	Am J Transplant/2010/open label RCT study	Basiliximab	1-EVL (1.5 mg/day)-low-dose CsA-Pred 2-evl (3 mg/day)-low-dose CsA-Pred 2-MMF-CsA-Pred	1.5 mg/day 3 mg/day	3–8 ng/ml 6–12 ng/mL	Lymphocele occurred in 6.6%, 11.2%, and 5.1% in low-dose EVL, high-dose EVL, and MMF, respectively. Impaired wound healing occurred in 1.8%, 4%, and 1.1% in low-dose EVL, high-dose EVL, and MMF, respectively Wound dehiscence occurred in 1.5%, 3.2%, and 1.5% in low-dose EVL, high-dose EVL, and MMF, respectively No comment on *p* value for either of the finding
Dantal et al. [10]	Transpl Int/2010/open label RCT study	Basiliximab/daclizumab	1-immediate EVL (Day 1)-CsA-Pred 2-delayed EVL (from week 5) MMF was given till week 5 along with CsA-Pred	0.75 mg twice a day adjusted to achieve 3–8 ng/mL	*C*0 = 3–8 ng/mL in both the groups	Wound healing complications were 40% immediate group and 37.8% delayed group at 12 months respectfully (*p* = 0.86 NS) Wound healing complications were 36.9% in immediate group and 33.8% in delayed group at 12 months respectfully (*p*-NS)
Cooper et al. [134]	Clin Transplant/2013/pool analysis of three RCT studies	----------------	1-EVL (1.5 mg/day)-CsA-Pred 2-evl (3 mg/day)-CsA-Pred 3-MMF-CsA- 1.5 or 3.0 mg or MPA, with CsA and steroids	1.5 mg/day 3 mg/day	------------------	Wound healing complication was 16.6% in 1.5 mg/day in EVL as compared to 14.3% in MMF (*p* = 0.255) But, it was 21.8% in EVL 3 mg/day significantly higher in MMF group (*p* < 0.001)
Cibrik et al. [135]	Transplantation/2013/open label RCT study	Basiliximab	1-EVL (3–8 ng/ml) + reduced exposure CsA + Pred 2- EVL (6–12 ng/ml) + reduced exposure CsA + Pred 3-MMF-standard CsA + Pred	Dose was adjusted to get level 3–8 ng/mL Dose was adjusted to get level 6–12 ng/mL	*C*0 = 3–8 ng/mlc0 = 6–12 ng/mL	Rare wound healing events of 0.4% in EVL (3–8 ng/mL), 0.7% in EVL (6–12 ng/mL), and 1% in MMF group
Chadban et al. [136]	Transpl Int/2014/open label RCT study	Basiliximab	1-CsA-MPS-Pred for 14 days, then EVL-Pred (CsA and MPS withdrawal) 2-CsA-mps-Pred for 14 days. then EVL-CsA (Pred and MPS withdrawal) 3-CsA-mps-Pred	Dose adjusted for first 15 to 60 days to achieve level 6–10 ng/mL From 61–120 days in CsA-MPA withdrawal group dose adjusted to level of 8–12 ng/mL. In Pred-MPA withdrawal group, EVL C0 level was kept at 6–10 ng/mL	15–60 days: both groups EVL C0 level = 6–10 ng/mL 61-120 days: CsA-MPA WD : EVL = CO = 8–12/ng/mL Pred-CNI WD : EVL = *C*0 = 6–10 ng/mL	Nonsignificant wound healing complication occurred in CsA-MPA WD (33%), Pred-MPA WD withdrawal (30%), and 32% in CsA-MMF-Pred
Nashan et al. [137]	Am J Transplant/2016/open label RCT study		Randomization after 10–14 weeks: 1-evl-mps-Pred 2-CsA/TAC-mps-Pred	EVL CO = 6–10 ng/mL	CO = 6–10 ng/mL	Wound healing events were similar in both the arms (EVR, 6.6 vs. CNI, 5.8%; *p* = 0.66 The incidence of patients with wound problem was similar in ≤25 percentile BMI category (EVR, 0.9 vs. CNI, 0.8%; *p* = 0.846). The incidence was higher in EVR arm in >25–≤50 (2.6 vs. 1.1%; *p* = 0.271) and significantly higher in >50–≤75 categories (2.0 vs. 0.6%; *p* = 0.049).
Qazi et al. [138]	Am J Transplant/2017/open label RCT study	Basiliximab or ATG	1-EVL-low-dose TAC-Pred 2-MMF-standard-dose TAC-Pred	EVR 0.75 mg twice a day (1.5 mg/day)	*C*0 = 3–8 ng/mL	Fluid collection (lymphocele, seroma, urinoma, and hematoma) adjacent to transplant was 22.5% (EVL) vs.15% (MMF) Delayed wound healing was 16.7% (EVL) vs. (11.8%)
de Fijter et al. [9]	Am J Transplant/2017/open label RCT study	Basiliximab	TAC or CsA-MPA-Pred for 10–14 weeks, then randomized to1-evl-mps-Pred 2-TAC or CsA-mps-Pred	Dose adjusted to CO level 6–10 ng/mL	*C*0 = 6–10 ng/mL	Wound healing events were similar between groups 5.8% (CNI) vs. 6.5% (EVL)
Ueno et al. [105], ATG	Transplantation/2017/subanalysis of open label RCT study	ATG/basiliximab	1-ATG followed by EVL-TAC-Pred 2-basiliximab followed by EVL-TAC-Pred 3-basiliximab followed by MPS-TAC-Pred	Dose adjusted to get level *C*0 = 4–8 ng/mL Dose adjusted to get level *C*0 = 4–8 ng/mL	*C*0 = 4–8 ng/mL C0 = 4–8 ng/mL	Basiliximab-EVL group has 35.2% wound healing complication vs. 22% in basiliximab-MPS (*p* = 0.033)
Tedesco-Silva et al. [69]	J Am Soc Nephrol/2018/Open label RCT study	Basiliximab/ATG	1-EVL-TAC or CsA-Pred 2-mmf or MPS –TAC or CsA-Pred	EVL dose was 1.5 mg or 0.75 mg twice a day in TAC or CsA	*C*0 = 3–8 ng/mL	Wound healing was 19.8% in EVL group as compared to 16.2% with relative risk 1.22 (1.01 to 1.47)
Sommerer et al. [139]	Kidney International/2019/open label RCT study	Basiliximab	1-EVL-TAC-Pred 2-EVL-CsA-Pred 3-MMF-TAC-Pred	EVL dose was adjusted to *C*0 = 3–8 ng/mL	*C*0 = 3–8 ng/mL	Wound healing was 30.5%, 28.3%, and 33.3% in the three groups, respectively, and did not differ in three groups. Lymphocele occurred in 18.1%, 24%, and 20.1% of the three groups, respectively
Manzia et al. [18], IR VS. DR	Transplantation/2020/open label RCT study	IL-2 receptor antibodies/ATG	1-immediate (IE) EVR-low CsA-Pred 2-delayed group (DE):MPS-CsA-Pred for 28 days followed by EVR-low CsA-Pred	EVL 0.75 mg BID	*C*0 = 3–8 ng/mL	Wound healing complications included in IE were fluid collection (17%), lymphocele (10%), and wound dehiscence (6%), which were not different at 3 months as compared to DE group At 12 months, wound healing complications between IE and DE groups were not significant except hematoma which was significant in IE group (*p* = 0.02380) as compared to DE and lymphocele was more in DE group as compared to IE group (P0.0368)

## Data Availability

This is a review article with no data.

## Abbreviations

Pred: Prednisolone
mTOR-I: Mammalian target of rapamycin inhibitors
ATG: Antithymocyte globulin
BMI: Body mass index
CNI: Calcineurin inhibitor
CsA: Cyclosporine
ER-TAC: Extended-release tacrolimus
MMF: Mycophenolate Mofetil
MPA: Mycophenolic acid
SRL: Sirolimus
TAC: Tacrolimus.
